# Predicting type 2 diabetes via machine learning integration of multiple omics from human pancreatic islets

**DOI:** 10.1038/s41598-024-64846-3

**Published:** 2024-06-25

**Authors:** Tina Rönn, Alexander Perfilyev, Nikolay Oskolkov, Charlotte Ling

**Affiliations:** 1 Epigenetics and Diabetes Unit, Department of Clinical Sciences, Lund University Diabetes Centre, Scania University Hospital, Lund University, 205 02 Malmö, Sweden; 2 Science for Life Laboratory, Department of Biology, National Bioinformatics Infrastructure Sweden, Lund University, Sölvegatan 35, 223 62 Lund, Sweden

**Keywords:** DNA methylation, RNA-sequencing, Genetic variation, Metabolic disease, Omics integration, Machine learning, Epigenetics, MultiOmics analysis, Insulin secretion, Beta-cell, EWAS, GWAS, Computational biology and bioinformatics, Genetics, Biomarkers, Diseases, Endocrinology, Medical research, Molecular medicine

## Abstract

Type 2 diabetes (T2D) is the fastest growing non-infectious disease worldwide. Impaired insulin secretion from pancreatic beta-cells is a hallmark of T2D, but the mechanisms behind this defect are insufficiently characterized. Integrating multiple layers of biomedical information, such as different Omics, may allow more accurate understanding of complex diseases such as T2D. Our aim was to explore and use Machine Learning to integrate multiple sources of biological/molecular information (multiOmics), in our case RNA-sequening, DNA methylation, SNP and phenotypic data from islet donors with T2D and non-diabetic controls. We exploited Machine Learning to perform multiOmics integration of DNA methylation, expression, SNPs, and phenotypes from pancreatic islets of 110 individuals, with ~ 30% being T2D cases. DNA methylation was analyzed using Infinium MethylationEPIC array, expression was analyzed using RNA-sequencing, and SNPs were analyzed using HumanOmniExpress arrays. Supervised linear multiOmics integration via DIABLO based on Partial Least Squares (PLS) achieved an accuracy of 91 ± 15% of T2D prediction with an area under the curve of 0.96 ± 0.08 on the test dataset after cross-validation. Biomarkers identified by this multiOmics integration, including *SACS* and *TXNIP* DNA methylation, *OPRD1* and *RHOT1* expression and a SNP annotated to *ANO1*, provide novel insights into the interplay between different biological mechanisms contributing to T2D. This Machine Learning approach of multiOmics cross-sectional data from human pancreatic islets achieved a promising accuracy of T2D prediction, which may potentially find broad applications in clinical diagnostics. In addition, it delivered novel candidate biomarkers for T2D and links between them across the different Omics.

## Introduction

The complexity of the human genome necessitates integration of several Omics, *i.e.* different layers of information on top of the DNA sequence, to get further insights in the pathogenesis of type 2 diabetes (T2D). Next generation sequencing (NGS) technologies have revolutionized T2D research and provided unique information about the disease with unprecedented depth and scale^1^. Importantly, genome-wide association studies (GWAS) showed that a large proportion of risk single nucleotide polymorphisms (SNPs) for T2D are associated with impaired insulin secretion^2^. Indeed, pancreas is the key organ for understanding T2D pathogenesis since insulin and glucagon secretion from pancreatic beta and alpha cells, respectively, largely control blood glucose levels. Large efforts have therefore been made to dissect the molecular mechanisms that contribute to impaired insulin and glucagon secretion from pancreatic islets in patients with T2D. These include genome-wide RNA-sequencing (RNA-Seq)^3–7^ and DNA methylation analysis^8–11^ in human islets from donors with T2D and non-diabetic controls, which identified candidate genes for the disease. DNA methylation, *i.e.*, the attachment of a methyl group to the DNA, is an epigenetic mark indicative of gene activity. As DNA methylation mainly occurs on the nucleotide cytosine, it is also dependent on SNPs^10,12^. The genetic and epigenetic codes are eventually connected to RNA transcription in each cell, comprehensively quantified by RNA-Seq. A combined analysis of these complementary Omics data from different layers of cell organization in human pancreatic islets may provide synergistic effects for the analytical modeling of the disease, thereby identifying novel candidates not possible to detect when analyzing each Omic individually. Therefore, integrating a variety of biological layers of information (Omics data) from human pancreatic islets is a promising approach in frontline T2D research.

The traditional statistical approach to find links between different biological/molecular mechanisms has been the univariate pair-wise correlations of Omics layers, *e.g.*, GWAS^2^, eQTL^3^ and mQTL^10^ studies, which has predominantly been concentrated on discovering candidate biomarkers that potentially could provide a better understanding of mechanisms linked to T2D. Despite its simplicity and interpretability, this approach may suffer from the lack of predictive capacity and the “missing heritability” problem^13^. Importantly, Machine Learning represents an alternative direction in data analysis that aims at optimizing prediction of disease, in our case T2D, and provides an outstanding application for clinical diagnostics^14–16^. A key goal of data integration using Machine Learning across several different Omics (multiOmics) is to achieve a more accurate diagnosis of current and prognosis of future disease events, respectively, compared with using data from a single Omic. Additionally, new biological biomarkers of T2D pathogenesis can be identified using the Machine Learning paradigm via multiOmics feature extraction because of the optimized predictive capacity of the integrative model.

The first aim of this study was to apply Machine Learning to integrate multiple sources of biological/molecular information (multiOmics), in our case including RNA-seq, DNA methylation, SNPs and phenotypic data. After exploring existing Machine Learning methods and careful model selection based on complexity and appropriateness for our data, we established a predictive Partial Least Square (PLS) Regression model for Omics integration. Secondly, we applied this multiOmics Machine Learning model in a carefully selected cohort of human pancreatic islets from donors with T2D (n = 32) and non-diabetic controls (n = 78). Of note, we found that this approach, first, achieved a very good accuracy of T2D prediction, that can potentially find applications in the clinical diagnostics, and second, delivered novel candidate biomarkers and links between them across the different Omics.

## Results

### Selecting a Machine Learning approach for multiOmics integration

First, we describe the idea of multiOmics analyses, including Omics integration, and explore the Machine Learning way of integrating multiple sources of molecular/biological information, in our case RNA-seq, DNA methylation, genotype (SNP) and phenotypic data. We present different Machine Learning methods available for multiOmics analyses as well as their pros and cons. Then, we describe and apply our selected model as well as the way we validate the success of data integration.

**Figure 1:**
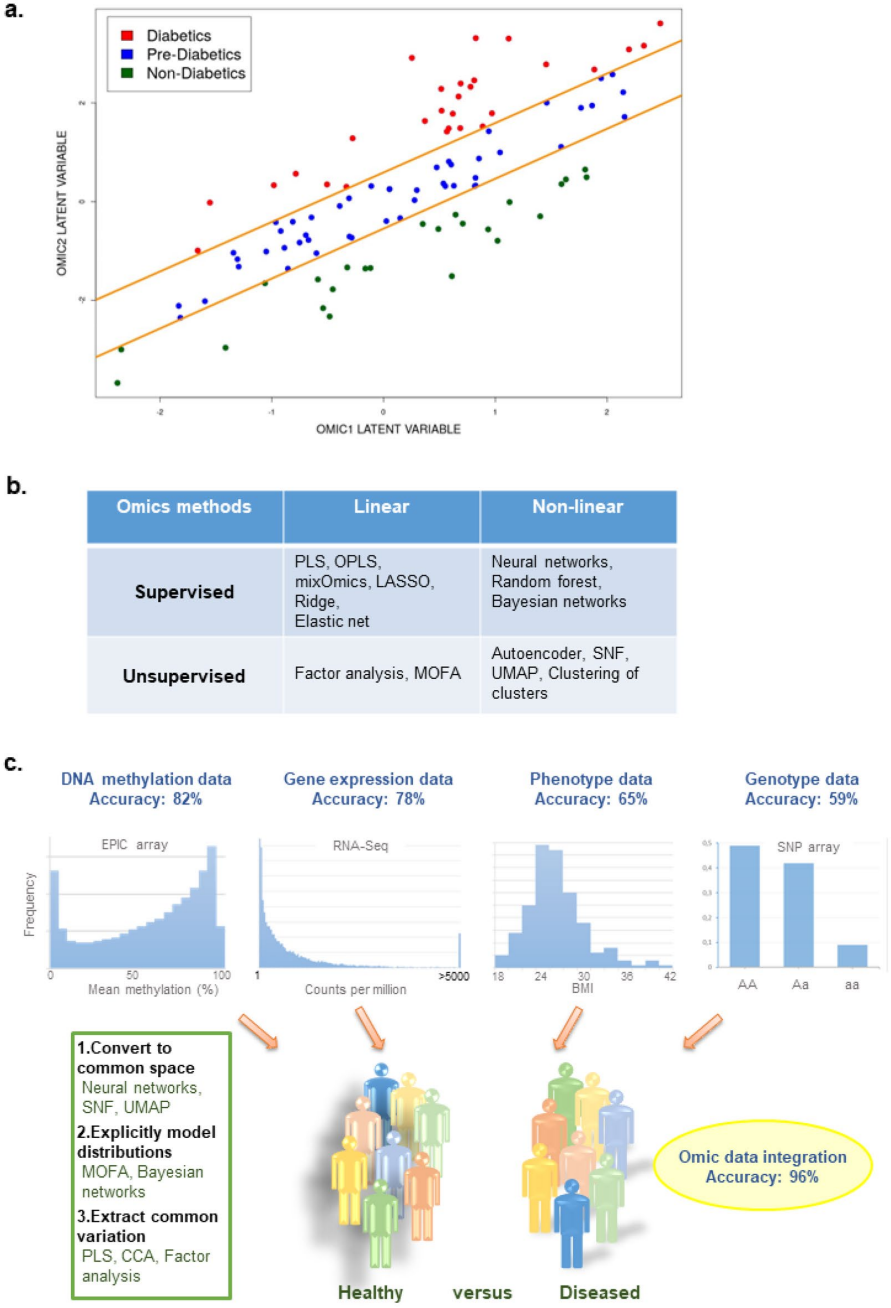
MultiOmics Integration Workflow. (**a**) Illustration of the idea and principle behind multiOmics integration: to see patterns hidden in individual Omics. The classes of data points cannot be reliably determined using separate Omics axes, however, become linearly separable when putting the Omics against each other. (**b**) Presents different Machine Learning methods for multiOmics integrations, including supervised linear methods such as Partial Least Squares (PLS) regression, Orthogonal Partial Least Squares (OPLS), mixOmics, Least Absolute Shrinkage and Selection Operator (LASSO), Ridge regression, and Elastic net regularization, supervised non-linear methods such as Neural networks, Random Forest and Bayesian networks, unsupervised linear methods such as Factor analysis and MultiOmics Factor Analysis (MOFA) as well as unsupervised non-linear models such as autoencoder, Similarity Network Fusion (SNF), Uniform Manifold Approximation and Projection (UMAP) and Clustering of clusters. The choice of integrative multiOmics method depends on (1) sample size and (2) presence of a phenotype of interest. In this study we prioritized a supervised linear method (PLS) since we have a limited number of samples and T2D as a clear phenotype of interest. (**c**) A schematic overview of the ambition of multiOmics integration to achieve a boost in the predictive capacity compared to the predictions of each Omic individually. Since the Omics data are sampled from very different underlying probability distributions (top of the figure exemplifies the distribution), we cannot simply concatenate the Omics into a single matrix without at least converting them to a common space where their technological memory is lost (left box).

By Omics integration using Machine Learning, we understand the ability of the model to deliver new biological knowledge that is not accessible in each individual Omic layer. This is schematically illustrated in a hypothetical example in Fig. 1a, where the data points belong to three different classes (in our hypothetical example non-diabetics, pre-diabetics and diabetics). The axes of the plot represent latent variables (*e.g.* Principal Components generated by Principle Component Analysis (PCA)) for two Omics. More specifically, the x-axis of Fig. 1a can be the first principal component (PC1) computed on Omic1, and the y-axis can be PC1 computed on Omic2. Projection of the data points on one of the axes does not provide an obvious separation between the three classes, as the points do not form distinct clusters. However, the way we depict the two Omics data points against each other via their individual latent variables, and the assumption that the Omics co-vary, *i.e.* demonstrate common variation, provides a way to linearly separate the three classes of data points. This idea should be applicable to any integrative model, provided that a descent level of linkage (co-variation) is present between the Omics.

There are several possible Machine Learning methods for Omics integration to choose from, *e.g.* the linear Partial Least Squares (PLS)^17^ via the Data Integration Analysis for Biomarker discovery using Latent cOmponents (DIABLO) algorithm^18^ or O2PLS^19^, as well as the non-linear Bayesian Networks^20^, Random Forest^21^ and Deep Neural Network (https://github.com/ueser/FIDDLE) models. In general, the choice of the integrative model depends on two factors: 1) amount of data, and 2) availability of phenotypes of interest. The human pancreatic islets dataset included in this study comprises relatively few samples (110 donors); therefore, a linear type of integrative analysis should be prioritized. Performing a non-linear analysis would involve many more fitting parameters and as a consequence the danger of overfitting the model, *i.e.* when the model is not generalizable and fails validation in an independent dataset. Moreover, since the phenotype of interest in our study, T2D, is a well-defined trait, we chose to perform a supervised classification task via multivariate extraction of biological features discriminating between diabetics and non-diabetics. Alternatively, if one assumes a hidden sub-structure in the data, *e.g.* more than two classes present (diabetics and non-diabetics), a variety of methods such as multiOmics Factor Analysis (MOFA)^22^, Similarity Network Fusion (SNF)^23^ and Deep Autoencoder^24^ are available for performing unsupervised Omics integration. However, here, we assume that all the heterogeneity in the data is captured within the binary classification problem and all individuals are assigned to either the diabetic or non-diabetic class. Figure 1b presents an overview of several possible linear/non-linear and supervised/unsupervised integrative Omics methods.

The goal of the Machine Learning approach to Omics integration was to build a model with a predictive capacity exceeding the ones from analyses of individual Omics. In other words, a successful integrative model on human pancreatic islet data should ideally demonstrate a boost in prediction of T2D compared to the predictions obtained from separate DNA methylation, gene expression, genetic variation (SNPs), and phenotypic datasets, as exemplified in Fig. 1c. Improved predictive capacity manifests the discovery of novel biological links via multivariate analysis of biomarkers across multiple layers of cell organization. However, to achieve increased accuracy of T2D prediction, it is not optimal to simply concatenate the data matrices from individual Omics, as they originate from different underlying statistical distributions. Various approaches have been suggested to solve this issue, generally falling within three categories: (1) convert individual Omics to a common parameter space where they lose the “memory” of technological differences (artificial neural networks, SNF, UMAP^25^), (2) explicitly model the individual Omics statistical distributions (MOFA, Bayesian Networks), and (3) extract common variation across individual Omics and factor it in order to interpret the common sources of variation (PLS, O2PLS, Canonical Correlation Analysis^26^, Factor Analysis, DIABLO^18^) (Fig. 1c).

Further, biological and biomedical Omics typically represent high-dimensional datasets with tens of thousands (gene expression) or even millions (DNA methylation and genetic variation) of features. This implies that any statistical analysis, including Omics integration, performed on the high-dimensional data will suffer from the Curse of Dimensionality^27,28^, *i.e.* inability to discriminate between diabetics and non-diabetics due to increasingly equal similarities between the data points in the high dimensional parameter space. To overcome this obstacle, we implement a feature pre-selection procedure for each individual Omic prior to the integrative analysis. Feature pre-selection can be performed in a supervised, *i.e.* providing the phenotype of interest, or unsupervised/unbiased fashion. The unsupervised way is typically based on selecting the most variable features regardless of the origin of variation. Therefore, features selected in this way often manifest high variation due to technical (such as batch-effect) and other non-biological reasons. To avoid that, we here prioritized the supervised feature pre-selection strategy which can be done for example via LASSO, Ridge or Elastic Net regression^29^, Linear Discriminant Analysis (LDA)^30^ or already mentioned PLS^17^. These algorithms are linear and supervised in nature, and use similar underlying assumptions; therefore, the feature pre-selection step should be robust regardless of the choice of a particular method. In the present study, we used the DIABLO algorithm^18^ for performing integrative multiOmics analysis (see below). Since DIABLO is a PLS based method, for consistency with the other steps of the integrative Omics workflow, we performed feature pre-selection via PLS as well.

Our choice of multiOmics integrative method was DIABLO, implemented within the mixOmics R Bioconductor package^18^. The analysis workflow developed and used for our multiOmics data pre-processing and integration is available at https://github.com/NikolayOskolkov/IntegrativeOmicsWorkflow. DIABLO generalizes the PLS approach to the case of multiple Omics datasets corresponding to the same samples (statistical observations). The idea of DIABLO is to transform each individual Omic dataset into latent components and maximize the sum of pairwise correlations between the latent components and a phenotype of interest. The result of DIABLO is the identification of features that are correlated between and within the Omics datasets. The choice of DIABLO was motivated by the fact that it is a linear and a supervised algorithm that fits the setup of our study that contains a relatively low number of samples and has a clear phenotype of interest (T2D). The advantages of DIABLO compared to other supervised integrative frameworks such as Random Forest and Artificial Neural Networks are its interpretability and relative simplicity, which helps avoiding overfitting. In addition, the implementation of DIABLO within the mixOmics R package provides informative visualizations of the decision boundaries between T2D and control individuals, driven by the selected features.

**Figure 2:**
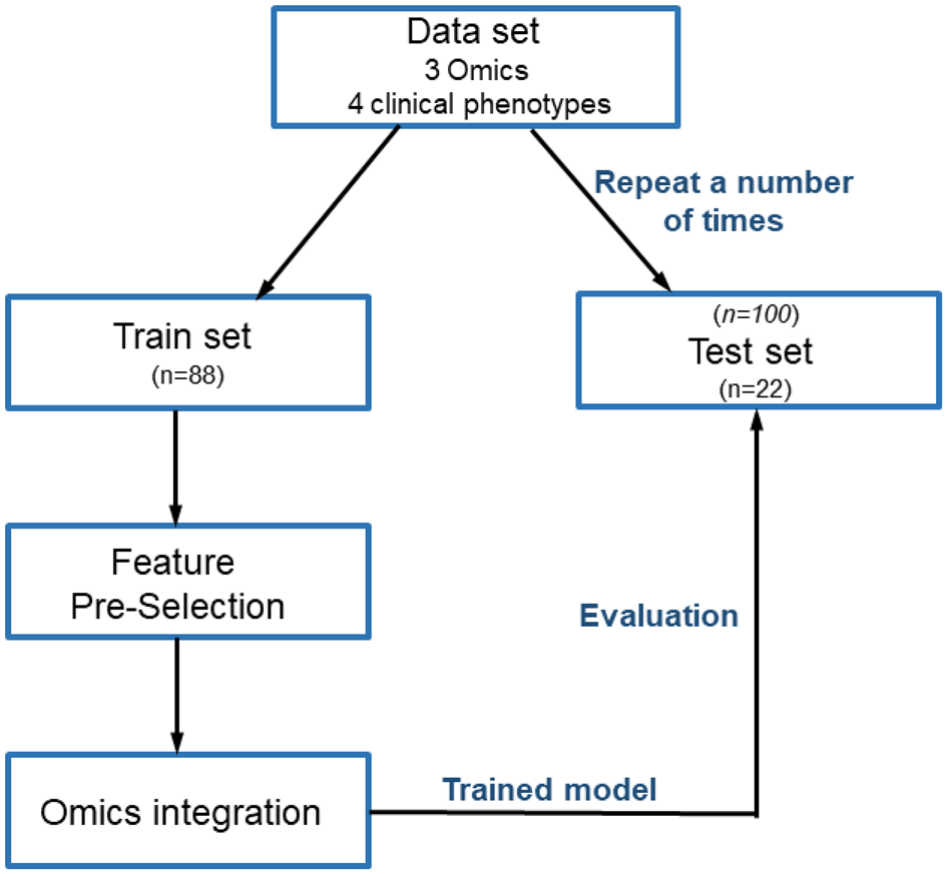
A schematic presentation of our integrative multiOmics analysis. The full dataset is randomly split into train (n = 88, 80%) and test (n = 22, 20%) datasets. Feature pre-selection and multiOmics integration are performed on the train set, and the model is evaluated on the test set. This procedure was repeated 100 times, and confidence intervals of the predictive model were built via the hold-out cross-validation strategy, *i.e.* multiple splitting of the data into train and test datasets.

Following a typical Machine Learning analysis strategy, we randomly split the 110 individuals with three Omics and clinical phenotypes (RNA-Seq, DNA methylation, SNP and phenotypic data) into train (80% of samples, 88 individuals) and test (20% of samples, 22 individuals) datasets. We then performed both feature pre-selection in the individual Omics and data integration across Omics using the train dataset, and then evaluated the model on the 20% test samples that were not used to train the model (Fig. 2). Since the key goal of the integrative multiOmics analysis is to optimize the prediction of T2D, we must incorporate uncertainty of prediction into our model, thereby achieving a more accurate modeling of the data. For this reason, we implemented multiple (*n* = 100) random splitting of the total 110 individuals into train and test datasets within the hold-out cross-validation framework. This allows building confidence intervals of the predictive integrative model (Fig. 2). Each split was performed in an unstratified way, i.e. without keeping the initial ratio of T2D cases and controls, which should result in wider confidence intervals and a more conservative and generalizable model. In other words, the model will have to learn to adapt to data sets with both many and few T2D cases. Finally, we compared the prediction results of the integrative multiOmics analysis with DIABLO against T2D prediction based on individual Omics.

Although, in this study, we use the DIABLO algorithm, as an example, to demonstrate the Machine Learning approach to Omics integration, we believe that the suggested approach of multiOmics analysis is generalizable for other Machine Learning methods. Therefore, as a summary and one of the key results of this paper, we here propose a procedure of Machine Learning multiOmics integration:

1. Choose an integrative model based on the amount of data (linear or non-linear) and the goal of the study (supervised or unsupervised) (Fig. 1b)
2. Perform feature pre-selection in the individual Omics using train dataset
3. Perform Omics integration according to the model of your choice (Fig. 1b)
4. Evaluate the prediction of the integrative model on the test dataset and compare with the predictions from individuals Omics (Fig. 1c)

The proposed procedure can help facilitate integrative multiOmics analysis for different types of data in biomedicine, bioinformatics, and data science.

### Applying the Omics integration procedure for type 2 diabetes prediction

We next applied this procedure of Machine Learning multiOmics integration to cross-sectional data from a human cohort that consists of high-quality pancreatic islets from 110 donors, including 32 T2D cases and 78 controls (Table 1).

**Table 1:** Phenotypes for the 110 donors of pancreatic islets, including 32 T2D cases and 78 controls.

Phenotype	T2D cases	Controls	*P*-value
Sex (male/female)	20 / 12	48 / 30	
Age (years)	62.9 ± 7.7	61.5 ± 8.0	0.40
BMI (kg/m^2^)	27.8 ± 3.8	26.0 ± 3.9	0.03
HbA1c (mmol/mol)	51.5 ± 9.7	36.7 ± 3.6	1.4 × 10^–19^
Stimulatory index (glucose stimulated insulin secretion)	6.3 ± 5.2	8.0 ± 7.0	0.23
Islet purity (%)	82.6 ± 7.9	82.9 ± 8.3	0.87

Data is shown as mean ± sd. *P*-values are based on two-sample t-tests (two-tailed).

**Figure 3:**
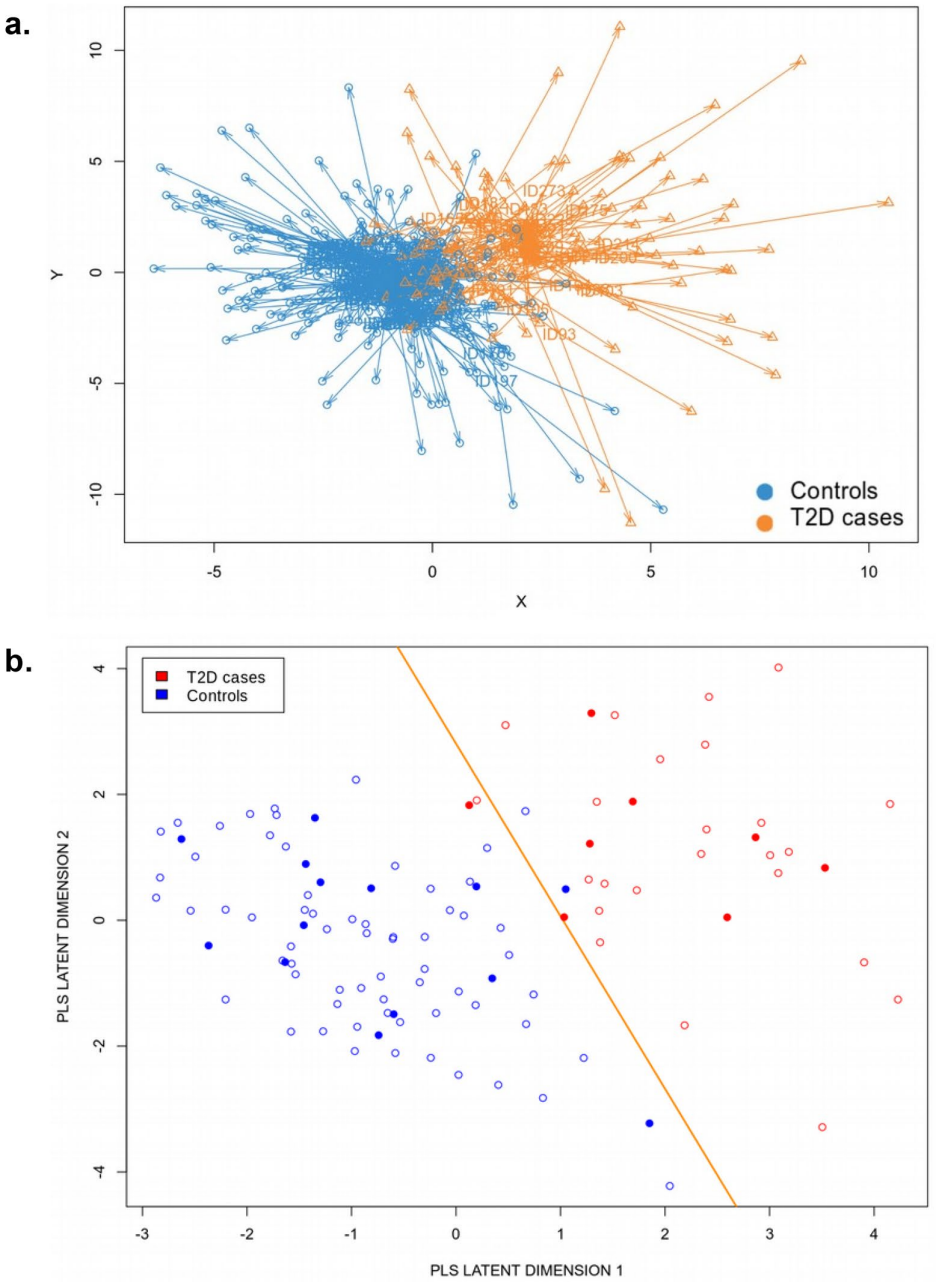
Type 2 diabetes (T2D) prediction. (**a**) Consensus across Omics PLS space visualization of 110 samples, including 32 T2D cases and 78 controls. Tips of the arrows demonstrate the position of samples according to individual Omics, while the centroids depict a consensus across the Omics position of each sample. (**b**) Demonstration of the linear decision boundary that DIABLO integrative Omics PLS classifier learns on the train samples (open circles), and projection of the test samples (filled circles) onto the latent PLS space that is consensus/averaged across the multiOmics.

By implementing a multiple hold-out cross-validation procedure, we first extracted a set of the most informative features in individual Omics persistently contributing to the prediction of T2D status across most of the train-test splits. This procedure was performed via PLS loading scores by ranking biomarkers by their importance for each train-test split, and included features appearing in at least 70% of the 100 train-test split iterations (Fig. 2, Supplementary Table 1). Constructed in this way, the final integration of top ranked features included expression of 38 genes, DNA methylation of 33 sites and three genotypes/SNPs, together with four clinical phenotypes (Table 2). This set of features/biomarkers was then used to visualize the 110 samples, including 32 T2D and 78 non-diabetic controls, in the latent DIABLO space as a consensus Arrow Plot across the three Omics datasets and the four phenotypes (Fig. 3a). Here, the tips of the arrows denote positions of the samples using the individual Omics data, while after integration each sample is placed in the centroid (average across Omics) which is the beginning of the arrows.

**Table 2:** Top ranked selected features included in the final integrative multiOmics model for T2D prediction.

Expression features	DNA methylation features		Genotype features		Phenotype features
Gene name	Illumina ID	Nearest gene/region *	SNP ID	Nearest gene/region	
*ARG2*	cg00970981	intergenic	rs13279576_A	*LOC101929294* / intronic	Age
*ARL4C*	cg02736232	*IRF8* / TSS1500	rs7931183_A	*ANO1* / intronic	Sex
*BARX1*	cg02966936	intergenic	rs7430710_A	intronic	BMI
*CACNG5*	cg02988288	*TXNIP* / 5'UTR			Stimulatory index (insulin secretion)
*CHL1*	cg03622758	*ZDHHC3* / TSS1500			
*CLTRN*	cg03770217	*FAM109A* / 5'UTR			
*CNTN5*	cg04255401	intergenic			
*COMP*	cg04577129	intergenic			
*CPXM2*	cg05627498	intergenic			
*DKK3*	cg06184251	intergenic			
*ELFN1*	cg07175985	*SACS* / TSS200			
*FOXE1*	cg08248985	*ROR1* / 5'UTR			
*FSTL4*	cg09216797	*INPP5A* / 5'UTR			
*FXYD2*	cg09449232	*RP11-266E14.1* / lincRNA			
*GABRA2*	cg09467248	intergenic			
*GAD1*	cg11515284	intergenic			
*GCNT4*	cg12164242	*NCOR2* / 5'UTR			
*GLRA1*	cg12220370	*RBFOX3* / 5'UTR			
*GRAMD2B*	cg12451325	*RP11-665G4.1* / antisense			
*HCN4*	cg13336515	intergenic			
*KCNA1*	cg13566279	intergenic			
*LRRC2*	cg13970113	intergenic			
*LSAMP*	cg14490520	intergenic			
*MPP1*	cg14527110	*P4HA2* / 5'UTR			
*NEFL*	cg14534405	intergenic			
*NIPAL4*	cg15630265	*SHANK2* / 3'UTR			
*NOTUM*	cg17826980	intergenic			
*OPRD1*	cg21165486	*MGST3* / 5'UTR			
*PCOLCE2*	cg21533994	*SLC15A4* / TSS200,3'UTR			
*PLA1A*	cg25934997	*SYNPO* / 5'UTR			
*PRELP*	cg25979005	intergenic			
*RASGRP1*	cg26445440	intergenic			
*REEP1*	cg26767974	*HDAC4* / 5'UTR			
*RHOT1*					
*SLC24A2*					
*SLC2A2*					
*SV2B*					
*TFCP2L1*					

These features were selected by PLS loading scores for ranking contributions of biomarkers, appearing in at least 70% of the 100 train-test split iterations.

*based on GENCODE basic v.12.

Essentially, the DIABLO integrative model classifier learns a linear decision boundary between individuals with T2D and non-diabetic controls, see Fig. 3b, where open circles represent consensus coordinates (centroids) of train samples across four Omics. Then, test samples can be projected onto the consensus latent DIABLO space by transforming their underlying Omics data in the same way as for the train samples, and the placement of the test samples (filled circles in Fig. 3b) with respect to the decision boundary provides the evaluation of the model’s performance.

**Figure 4:**
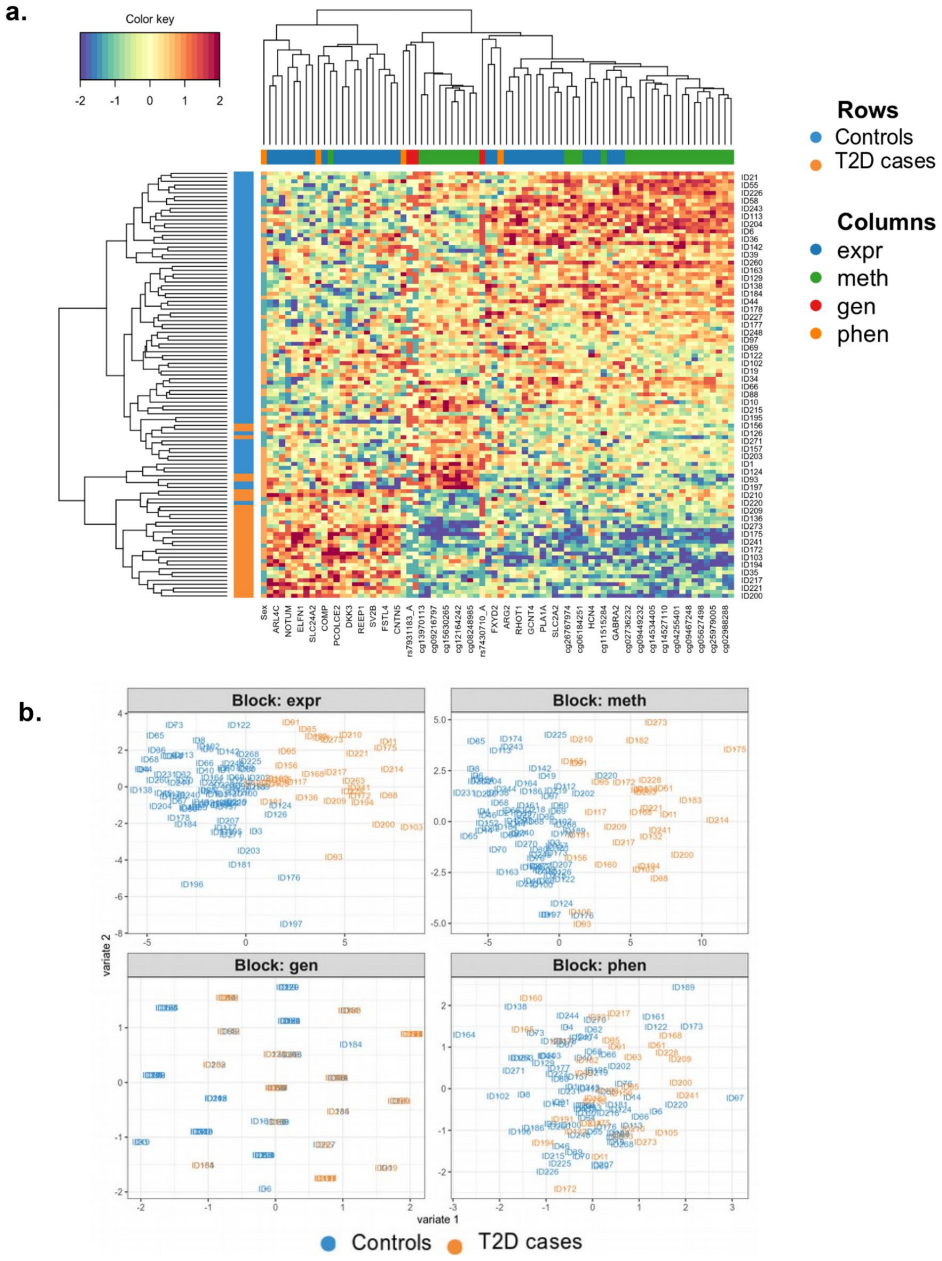
Contribution of individual Omics in a predictive model. (**a**) Heatmap displaying the results of hierarchical clustering on features selected via multiOmics integration. Individuals with type 2 diabetes (T2D, n = 32) and non-diabetic controls (n = 78) demonstrate a descent separation, while biomarkers responsible for that separation are inter-mixed with each other, reflecting the importance of interaction from all three Omics (mRNA expression (exp), DNA methylation (meth), genetic variation/SNPs (gen)) as well as clinical phenotypes (phen). (**b**) Contributions from the three individual Omic datasets (mRNA expression (expr), DNA methylation (meth), and genetic variation/SNPs (gen)) and the clinical phenotypes (phen) into the consensus integrative predictive model.

The consensus plots (Fig. 3a, b) demonstrate a remarkable linear separation of individuals with T2D and non-diabetic controls. One may subsequently expect to reach a high accuracy of T2D status prediction on the test set by converting the Omics data into PLS space and overlapping their coordinates across the Omics. Indeed, when computing the accuracy of T2D prediction on the test set for two DIABLO PLS components using 100 train-test hold-out cross-validation splits, we obtained 86.76 ± 14.59% (*p* = 0.03) accuracy for first and 90.52 ± 14.71% (*p* = 0.01) for second component (Supplementary Fig. 1). Since there are 32 T2D cases out of the 110 individuals in the cohort, *i.e.,* 29% have T2D, the dataset is slightly imbalanced and a naïve model that learns only the majority class, *i.e.,* that predicts every given individual to be a non-diabetic, would reach 71% accuracy of T2D versus control classification. Therefore, all the individual Omics models as well as the integrative model should give an accuracy significantly higher than the naive baseline 71%. For the DIABLO integrative multiOmics analysis, both components predict T2D significantly better than the naïve baseline model, and for PLS2 none of the splits resulted in a T2D prediction accuracy lower than 70%. The high accuracy of T2D versus control classification was confirmed by performing unsupervised hierarchical clustering on the top ranked features selected across the Omics bases on data integration (Fig. 4a). Notably, T2D and control donors cluster almost separately as the rows of the heatmap in Fig. 4a. This separation is driven by a combined effect from multiple features across all the three Omics and the four phenotypes, and not a particular Omic, as the columns of the heatmap, representing different features, are well inter-mixed.

By splitting the consensus plots (Fig. 3a, b) into contributions from individual Omics, we observed that gene expression and DNA methylation data strongly contribute to the final integrative model, while the clinical phenotype and genotype data seem to provide a minor contribution to the resulting separation between T2D and control samples (Fig. 4b). Nevertheless, the biomarkers selected via Omics integration seem to almost perfectly separate individuals with T2D and non-diabetic controls in the latent PLS space for each individual Omic (Fig. 4b) as well as in the consensus plot (Fig. 3a, b).

Looking at the most informative clinical phenotypic features, we discovered body mass index (BMI) driving the first and sex affecting most of the second PLS component (Supplementary Table 1). Despite being known to be linked with the risk of T2D and islet function^31,32^, these two phenotypes did not demonstrate significant correlation with T2D in our study, and no clear separation of T2D and controls in the PLS latent space for these phenotypes was observed in Fig. 4b. Yet, the integrative model suggests BMI and sex to be more important than the other phenotypes (age and stimulatory index) in predicting T2D.

**Figure 5:**
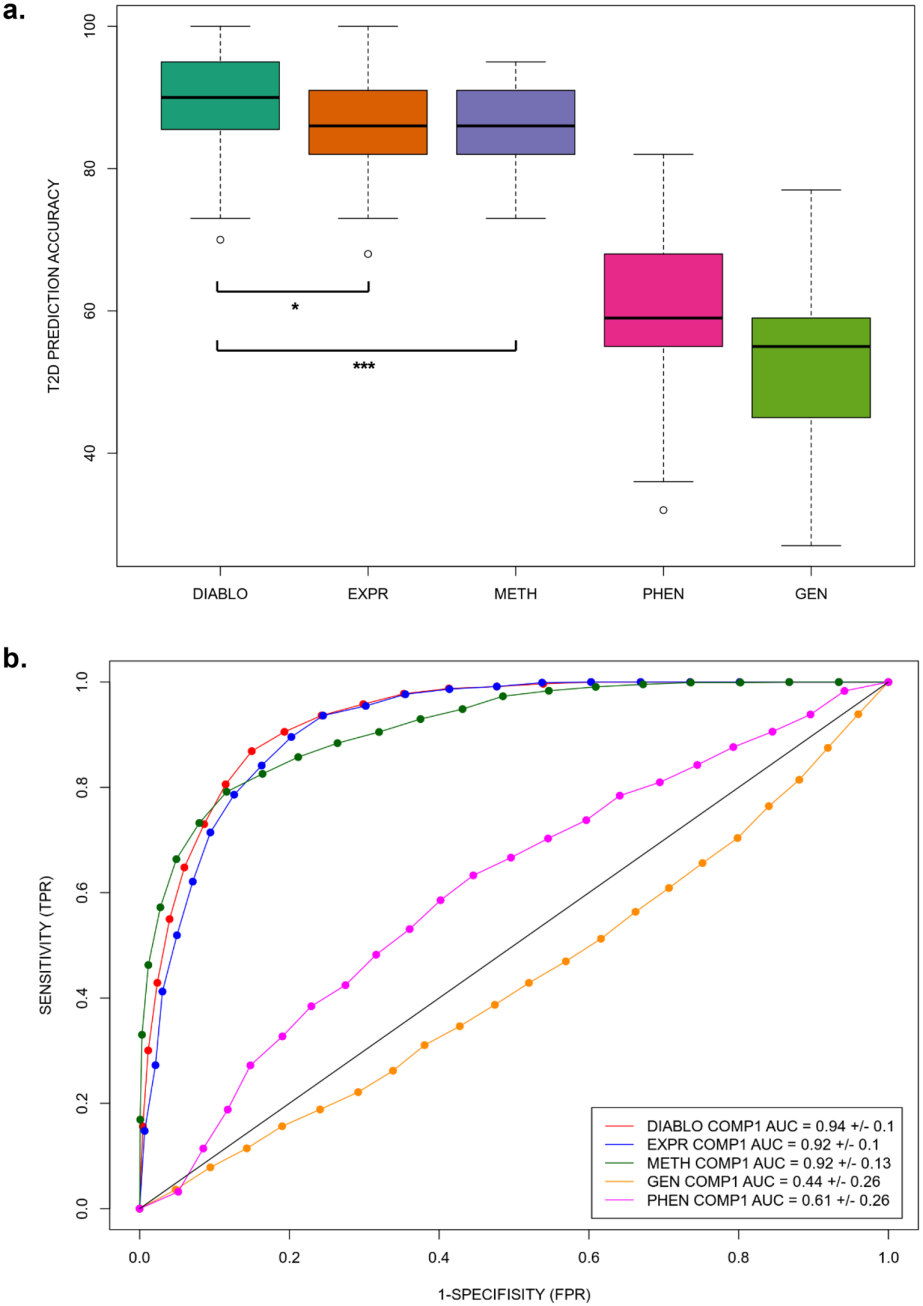
DIABLO prediction. (**a**) Comparison of type 2 diabetes (T2D) prediction accuracy of the integrative multiOmics DIABLO model (component 2) with the predictive capacities of the selected features of respective individual Omics (mRNA expression (expr), DNA methylation (meth), genetic variation/SNPs (gen)) as well as clinical phenotypes (phen). Sig. *: Mann-Whittney U test *p* = 0.02, Sig. ***: Mann-Whittney U test *p* = 0.00017. (**b**) Comparison of T2D prediction in the form of receiver operating characteristic (ROC) curves for the integrative multiOmics DIABLO and individual Omics models (mRNA expression (expr), DNA methylation (meth), genetic variation/SNPs (gen)) as well as clinical phenotypes (phen) (PLS component 1). The ROC area under the curve (AUC) for the multiOmics DIABLO model was significantly higher than both the ROC AUC for expression and the ROC AUC for DNA methylation with Mann-Whittney U test *p* = 0.01.

To further assess the contributions to the integrative DIABLO model from the selected features of the individual Omics, we built and evaluated PLS Discriminant Analysis (PLS-DA) models on the individual 38 RNA-Seq features, 33 DNA methylation features, three SNPs and four phenotypic datasets via a multiple hold-out cross-validation procedure (Fig. 5a). The results demonstrated that the RNA-Seq and DNA methylation datasets alone can provide a high prediction accuracy of T2D, 84 ± 13% (component 1) and 88 ± 14% (component 2) for gene expression, and 83 ± 13% (component 1) and 86 ± 12% (component 2) for DNA methylation. In contrast, the genotype and phenotype datasets alone demonstrated a poor predictive capacity, 52 ± 19% (component 1) and 51 ± 20% (component 2) for the SNP data and 60 ± 19% (component 1) and 60 ± 20% (component 2) for the phenotypic dataset, which is below the prediction accuracy of the naïve baseline model (71%). The failure of the genetic variation and phenotypic models may be explained by overfitting due to the limited sample size of our human pancreatic islets data. Despite the potential overfitting that led to non-optimal feature selection from those two Omics/phenotypes, certain phenotypes (*e.g.,* BMI) and genetic variants still seem to be informative and contributing to the integrative consensus T2D and control separation on the heatmap (Fig. 4a). From the results presented in Fig. 5a, we conclude that although integrative multiOmics DIABLO prediction marginally outperforms the predictions from individual Omics, it is driven largely by the RNA-Seq and DNA methylation data sets. Nevertheless, the integrative DIABLO analysis resulted in significantly higher accuracy compared to the individual gene expression data (Mann-Whittney U test *p* = 0.02), and the individual DNA methylation data (Mann-Whittney U test *p* = 0.00017), analyses.

It is also important to mention that the top ranked / predictive features obtained from the DIABLO model on individual Omics did not entirely overlap with the informative features identified by the integrative multiOmics DIABLO model. In fact, only expression of 11 genes, methylation of 10 CpG sites and 2 SNPs were present among the most predictive features picked by the DIABLO model executed on individuals Omics (Supplementary Table 2). Hence, despite that the integrative DIABLO model was driven to a large extent by gene expression and DNA methylation, several (approximately ~ 50%) features identified by DIABLO can be considered as novel in sense that they would not have been detected as most informative when running predictive analysis on individual Omics. Overall, this demonstrates the power of the integrative multiOmics DIABLO analysis, not only in terms of a better predictive model, but also based on the ability to discover novel biomarkers which potentially would have been missed in the analyses of individual Omics.

Despite accuracy is a very intuitive and interpretable metric for evaluating our integrative multiOmics model (fraction of successful predictions of the model), it is not fully optimal due to the imbalance between T2D and control samples in the dataset. A receiver operating characteristic (ROC) curve that represents a balance between sensitivity and specificity of predictions is widely used for evaluation of a predictive model. We therefore constructed ROC curves for both PLS predictive components for the integrative multiOmics DIABLO model as well as predictive models from the selected features of the three individual Omics and the four clinical phenotypes (Fig. 5b). Again, we observed a marginal increase in prediction and certainty of the integrative multiOmics DIABLO model, with the area under the ROC curve of 0.94 ± 0.10 (component 1) and 0.96 ± 0.08 (component 2). In comparison, the ROC curve for the selected RNA-Seq features was 0.92 ± 0.10 (component 1, significantly lower than the DIABLO ROC AUC for component 1 with Mann-Whittney U test *p* = 0.01) and 0.96 ± 0.08 (component 2), for the selected DNA methylation features was 0.92 ± 0.13 (component 1, significantly lower than the DIABLO ROC AUC for component 1 with Mann-Whittney U test *p* = 0.01) and 0.93 ± 0.14 (component 2), for the selected SNPs/genetic variation was 0.44 ± 0.26 (component 1) and 0.41 ± 0.27 (component 2), and for the clinical phenotype data was 0.61 ± 0.26 (component 1) and 0.61 ± 0.25 (component 2). As for the assessment of prediction accuracies, the ROC curves also indicate that the integration multiOmics DIABLO model is driven largely by the RNA-Seq and DNA methylation datasets. We further addressed the imbalanced T2D versus controls classification via comparison of the Matthew’s Correlation Coefficients (MCC)^33^ across the different models (Supplementary Fig. 2). MCC showed almost identical patterns as for the accuracy comparison presented in Fig. 5a, implying that the imbalance in our labels does not substantially affect the conclusions of our analysis.

### Biological interpretation of the Omics integration in human pancreatic islets

**Figure 6:**
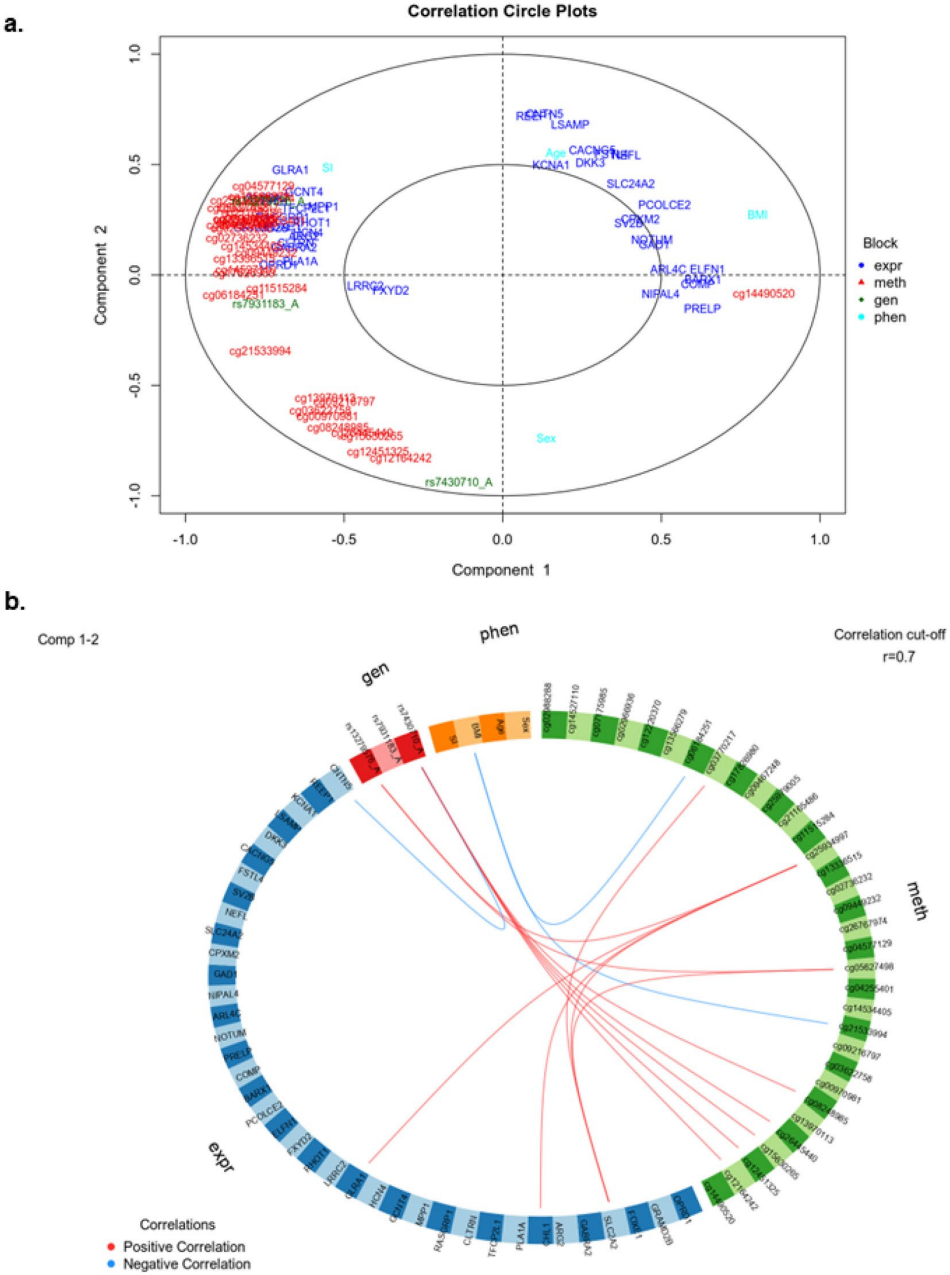
Biological interpretation. (**a**) Circle plot demonstrating the most predictive features located at the poles of the circle and between-features connections across the three Omics (mRNA expression (expr), DNA methylation (meth), genetic variation/SNPs (gen)) as well as clinical phenotypes (phen). (**b**) Circos plot demonstrating what features across Omics (mRNA expression (expr), DNA methylation (meth), genetic variation/SNPs (gen)) as well as clinical phenotypes (phen) that are most tightly linked in a presumable biological pathway.

Finally, to explain the high predictive capacity of the model, we visualized the features/biomarkers across the three Omics and the four clinical phenotypes that contribute the most to the prediction of T2D (presented in Table 2), as well as the relations between them. First, we constructed a Circle plot that overlaps the leading biomarkers/features across the three Omics and the four clinical phenotypes on a single circular plot, where features placed at the poles of the circle are most predictive for the integrative multiOmics DIABLO model, in contrast to the features in the center of the circle that are poor predictors of T2D. In addition, features across different Omics that happened to be located close to each other at the poles of the circle do also demonstrate some biological relation, as close proximity of features at the poles of the Circle plot implies strong correlations (Fig. 6a). For example, the proximal locations of the BMI phenotype and *BARX1* gene expression can imply their functional association. Importantly, most features aggregate close to the stimulatory index (SI) feature, including expression of 17 genes, DNA methylation of 22 sites and two genotype features (Fig. 6a), supporting their potential connection to glucose-stimulated insulin secretion measured by SI.

Secondly, we demonstrated connections between features across the three different Omics and the four clinical phenotypes in a Circos plot, where all features selected via DIABLO multiOmics integration are correlated with each other and the strength of the correlation can provide most linked features across the Omics (Fig. 6b). From the Circos plot, we observed strong correlations of DNA methylation levels of many CpG sites with both gene expression levels (*SLC2A2*, *CHL1* and *GLRA1*) and two genotypes (rs13279576_A and rs7430710_A). BMI was negatively correlated with DNA methylation of two CpG sites, supporting that even if the phenotypes alone are not a major driver of the DIABLO integrative multiOmics model, there is still interaction between Omics where the effect of BMI is seen first when connected to additional features, e.g., DNA methylation. Finally, we split the Circos plot into pairwise correlation network plots for each pair of Omics (Supplementary Fig. 3a–f), where the interaction network of gene expression and DNA methylation appears to demonstrate two disjointed modules with relatively high edge density, i.e. 8 edges per node on average (Supplementary Fig. 3a), which stands out compared to the network edge densities of other pairs of Omics, 1–2 edges per node on average (Supplementary Fig. 3b–f). Such interaction-rich network suggests that the two Omics cooperatively contribute to the prediction of the integrative model. The three identified SNPs seem to interact independently of each other with different sets of gene expression and DNA methylation features (Supplementary Fig. 3b, d). Among the clinical phenotypes, BMI has most connections with gene expression and DNA methylation features (Supplementary Fig. 3c, e).

Exploring the top of the lists of the most predictive features across the three Omicss (Table 2 and Supplementary Tables 1, 2), we noticed several interesting biomarkers. First, the methylation levels of a *TXNIP* 5’UTR CpG site (cg02988288) is a main T2D predictor of component 1 of the integrative multiOmics DIABLO model, a site previously associated with subgroups of T2D based on DNA methylation levels in blood^34^. Also, DNA methylation of cg07175985, located within 200 bp from transcription start site of *SACS*, is one of the main contributors to the prediction of T2D in our integrative multiOmics DIABLO model according to the ranking in the Supplementary Tables 1, 2. Gene expression of *SLC2A2* and *CHL1* are also among the main drivers of DIABLO PLS component 1, and interestingly, *SLC2A2* has previously been shown to have a role in development of neonatal diabetes^35^ and both genes’ impact on insulin secretion has been investigated^3^. Moreover, *OPRD1* expression, that is an additional key contributor to component 1 of the integrative multiOmics DIABLO model, has been associated with Alzheimer’s decease^36^, and it was recently shown that downregulation of this gene in human islets impact insulin secretion^3^. Interestingly, the *RHOT1* (Mitochondrial Rho GTPase 1) gene*,* which is related to mitochondrial function and insulin secretion^11^*,* was not top ranked when analyzing the gene expression Omic alone, but was highly ranked as very predictive by our integrative multiOmics DIABLO model.

In conclusion, the current integrative multiOmics approach identified several novel associations with T2D and confirms some previously identified genes and CpG sites. In addition, this integrative approach enhances our understanding of the interplay of different layers of biological information in the T2D pathology.

## Discussion

Here, advanced Machine Learning methods were applied to best integrate and explore the complex combination of gene expression, DNA methylation, genetic variants, and clinical phenotypic data in the T2D pathogenesis. Importantly, this is the first study to integrate three Omics and several clinical phenotypes from the same donors of human pancreatic islets into one analysis, and this innovative design revealed novel target regions for understanding islet biology and future treatment of the disease.

Biomedical Big Data from different sources (Omics data) can have synergistic effects, which may allow better modeling the behavior of biological cells and tissues. In this way, multiOmics integration can identify novel biological mechanisms and pathways that are not necessarily distinguishable in the separate Omics layers. Further, new mathematical methodologies are needed to deal with Big Data, among them Artificial Intelligence (AI) and Machine Learning are ideally suited for processing and integration of large amounts of data as well as generating predictive models that can potentially be used in *e.g.,* clinical diagnostics within the concept of precision medicine. In the present study, we used cross-sectional data to make "diagnostic predictions", i.e. train a model on multiOmics data from one group of individuals, and then take another group of individuals with similar data types and assess their T2D risk at one time point. In a different study, we previously showed that blood-based DNA methylation biomarkers associated with future T2D and insulin secretion in prospective cohorts, mirror the DNA methylation pattern in human pancreatic islets^37^, suggesting that we may also be able to follow diabetes-associated expression and methylation changes in human islets based on epigenetic biomarkers in blood. Still, studies of human complex diseases integrating several Omics remain scarce. Nevertheless, human pancreatic islet gene expression in combination with plasma lipidomics revealed potential prognostic markers for increasing HbA1c^6^. Additionally, Lawlor et al. combined several Omics to profile EndoC-βH1, a human beta cell line^38^. Integrating genetic, epigenetic, transcriptomic and chromatin data from this cell line provided a well-defined tool for mechanistic studies of the insulin secreting cell. However, studies like ours, demonstrating differences between pancreatic islets from individuals with T2D versus normoglycemic individuals are vital to discover the genes and genomic regions of importance for the disease.

The circos plot and pairwise correlation networks are examples of how multiOmics integration gives a deeper understanding than interpreting data from each Omic individually, as gene regulation and cellular function are results of complex interactions involving more than one feature and more than one Omic. For example, we observed strong correlations between DNA methylation of several CpG sites and gene expression of *SLC2A2*, *CHL1* and *GLRA1.* Expression or genetic variation of those genes are all connected to T2D pathogenesis and/or insulin secretion^3,35,39^, but based on our findings these results should ideally also be integrated with epigenetic marks.

As a strength and confirmation of our current integrative multiOmics approach, we were able to identify several known T2D candidate genes (*e.g., CHL1*, *SLC2A2* and *TXNIP*), found in studies of a single Omic^3,9,34,35^, but also several novel discoveries. Among the known genes, *TXNIP* has repeatedly been linked to T2D, it is induced by glucose in human pancreatic islets and elevated in T2D subjects, leading to beta cell dysfunction and apoptosis^40^. Moreover, *TXNIP* DNA methylation in blood has been associated with T2D subgroups and in particularly severe insulin deficient diabetes (SIDD)^34^. From the current study, we can extend these findings with differential DNA methylation of *TXNIP* also in islets from T2D donors. One interesting finding was the differential DNA methylation of *SACS*, a gene expressed in several cell types and required for normal mitochondrial dynamics^41^. Although this gene has not been studied in beta cells specifically, knock-down of *SACS* in another human cell type was shown to affect genes involved in oxidative phosphorylation (OXPHOS) and oxidative stress, and to induce impaired mitochondrial bioenergetics^41^. As human pancreatic islets from T2D donors display a reduced expression of genes impacting oxidative phosphorylation^42,43^, *SACS* may be epigenetically targeted in beta cells in a future attempt to restore mitochondrial function and modify insulin secretion. Differential *OPRD1* gene expression in islets from T2D donors, as previously described^3^, was confirmed in our integrative multiOmics approach and found located in the cluster of features related to stimulatory index, a measure of insulin secretion, in the Circle plot. *OPRD1* encodes the delta-opioid receptor, important for cognitive function and regulating reward pathways, and has previously been associated with Alzheimer’s disease and opioid addiction^36,44^. Interestingly, its expression is affected by both genetic variants and altered promoter DNA methylation^36,44^, supporting the importance of studying several Omics when dissecting disease pathogenesis. Moreover, a genetic variant in *OPRD1* has been associated with obesity and appetite regulation, and this locus interacts with sex^45^. We also identified *BARX1*, which is a gene known to be associated with adiposity traits such as Waist Hip Ratio (WHR) and BMI^46^, and also displays higher expression in human islets from donors with T2D versus non-diabetic controls^3^.

Interestingly, the clinical phenotypes alone had a small predictive effect, and the effect of e.g., BMI was seen first when connected to additional Omics, in the integrated multiOmics DIABLO model, where BMI was associated with DNA methylation of two sites. Indeed, differential DNA methylation may be secondary to BMI, but there is also evidence for methylation sites with a causal effect on BMI^47,48^.

This study has some potential strengths and limitations. The study design includes carefully selected human islets of high purity, obtained from 32 individuals with T2D and 78 normoglycemic controls. The case and control groups in the current study were of similar age and had a small significant difference in BMI, while in the general population of T2D individuals would be older and have a higher BMI than non-diabetics. Selecting individuals is not optimal for generalization of the model, but facilitates learning informative features, which was the aim of this multiOmics approach. The pancreatic islets included in this study were from multiorgan donors, which were brain dead and kept alive in a respirator during surgery. We cannot exclude that this may affect the RNA and/or DNA. However, since the pancreas is a “sensitive” tissue due to its digestive enzymes, which may initiate pancreatitis if pancreas biopsies are taken, it has been ethically difficult to take biopsies from the pancreas of living people for research purposes. Subsequently, it has been difficult to compare the RNA expression and DNA methylation patterns in pancreatic islet samples taken from living people with the once taken from multiorgan donors, where tissues often are used for transplantation. Nevertheless, a previous study of the brain by Ervin et al. suggests that postmortem delay has minimal effect on the RNA integrity^49^. Additionally, in the present study we only used pancreatic islets from the Scandinavian transplantation unit in Uppsala, where all islet isolations are done according to a “islet transplantation protocol”, a very stringent protocol, and both islets from individuals with T2D and the controls were treated in the same way. Moreover, in our hands the islets behave “normal”, e.g., they respond to high glucose with increased insulin secretion^50^, apoptosis is low in control islets and can be initiated by glucolipotoxicity^50^, and we have been able to reproduce both DNA methylation and RNA expression data in different studies including different islet donors^3,8,9,11^. Additionally, several of our DNA methylation and RNA-seq data from the islets make sense “from a physiological point of view”, for example we identified increased DNA methylation and decreased expression of *INS* and *PDX1* in islets from T2D cases versus controls^8,9,51,52^. Subsequently, we believe the human islets used in our study represent a good model.

The limited sample size of pancreatic islet donors is a limitation which may affect the generalizability of our analysis. Hence, to minimize the risk of overfitting, we used cross-validation for the prediction. We also prioritized simple linear models (instead of complex non-linear models), which have the minimal number of fitting parameters and thus are least prone to overfitting. Moreover, at each step of our analysis, we tried to apply randomization and re-sampling strategies as well as multiple validation and cross-validation iterations to optimize the data usage. We also carefully computed confidence intervals via multiple train-test splits in unstratified ways making sure that the final conclusions of the analysis are informed by the uncertainties imposed by the limited data. Finally, we applied feature pre-selection on each individual high-dimensional Omic (i.e. we reduced dimensionality of each individual dataset prior to integration) to minimize the effect of the Curse of Dimensionality and thus the risk of overfitting. In summary, we believe that despite the limited data, we implemented several approaches to make as accurate and robust analysis as possible.

## Conclusions

Here, we have demonstrated the proof-of-concept Machine Learning integration applied to three molecular Omics and one clinical phenotypic dataset from human pancreatic islets. We implemented a supervised linear PLS DIABLO integrative method and achieved remarkable accuracy as well as a high area under the ROC curve of T2D status prediction on 110 individuals with almost 30% of diabetics. Our model not only demonstrates a state-of-the-art T2D predictive capacity that can potentially be implemented for clinical diagnostics but also provides novel biomarkers such as *SACS* DNA methylation that we recently linked to diabetes pathogenesis^11^. In addition, cross-links between different biomarkers across the different Omics were delivered as a result of the integrative model. This integrative multiOmics analysis increases our knowledge about the disease and may further advance diagnosis of T2D.

## Methods

### Aim, design and setting of the study

Our aims were, first to explore Machine Learning to integrate multiple sources of biological/molecular information (multiOmics), in our case including RNA-seq, DNA methylation, SNPs and clinical phenotypic data. Then to establish a predictive Partial Least Square (PLS) Regression model for Omics integration and apply this multiOmics Machine Learning model in a carefully selected cohort of human pancreatic islets from donors with T2D (n = 32) and non-diabetic controls (n = 78).

### Characteristics of participants and description of materials

Human pancreatic islets from postmortem donors were provided by the Human Tissue Lab (HTL) at Lund University Diabetes Centre (LUDC) and the Nordic Network for Clinical Islet Transplantation (Uppsala). Islets from multi-organ donors were prepared by enzyme digestion and density gradient separation and islet purity was measured by dithizone staining. Only islet preparations with a purity of 70% or more were included. Islets picked under microscopy (*n* = 3) were assigned a purity of 85%. A sample was considered a T2D case if at least one condition was fulfilled: 1) the donor was diagnosed with T2D, 2) the donor had a Hemoglobin A1c (HbA1c) ≥ 48 mmol/mol. For controls to be included, their HbA1c should be < 42 mmol/mol. The controls were also selected to be in the same age range (43–81 years) and have the same range of days in culture (DIC: 1–7) as the T2D cases. In total, islets from 110 donors were included in the analysis, 32 T2D cases and 78 controls (Table 1). This filtering results in almost every third sample to be a T2D case, which makes the dataset relatively balanced and appropriate for a Machine Learning framework.

For the integrative multiOmics analysis, we kept sex, age, BMI and stimulatory index (a measure of insulin secretion from the pancreatic islets) phenotypic variables. The phenotypes did not go through the feature pre-selection procedure because of low amounts of features. The HbA1c variable was excluded from the analysis due to its strong dependency with T2D status. Data from the individual Omics have partly been included in previous publications^3,10,11^.

### DNA methylation

DNA methylation was analyzed using the Infinium MethylationEPIC v1 array (Illumina, San Diego, CA, USA) as previously described^11^. The DNA methylation array data were processed using the lumi pipeline^53^, applying background fluorescence subtraction, correcting for dye-bias from the use of two-color channels and quantile normalization followed by BMIQ normalization for correcting the technical differences between the Type I and Type II probe types^54^. The methylation levels of all CpG sites were coded in the form of M-values^55^. The final DNA methylation dataset comprised 816,790 CpG sites from 110 individuals.

### RNA sequencing

Gene expression data were obtained from RNA sequencing with the use of Illumina HiSeq 2500, stranded library protocol and counting the reads aligned to hg38 human reference genome using Salmon^56^. The complete RNA-Seq procedure has been previously described^3^. DESeq2 normalization was used for eliminating technical biases due to difference in library sizes^57^. Lowly or non-expressed genes having an unnormalized median read count across all the samples less than 1 were removed from the analysis as likely non-informative features. The final gene expression dataset comprised 18,023 genes from 110 individuals.

### Genotyping

Genome-wide SNP data from the 110 donors were obtained using the HumanOmniExpress genotyping array (Illumina). The data pre-processing steps included standard quality control procedures^58^ using PLINK^59^, *e.g.,* sample quality control via missingness versus heterozygosity assessment, sex, relatedness and population structure checkups, as well as genetic variant quality control via variant missingness, Hardy–Weinberg equilibrium and filtering out monomorphic variants. The final genotype dataset comprised 222,834 genetic variants from 110 individuals.

### Statistical analysis as well as software and bioinformatic tools

The integrative multiOmics analysis was performed using a block-PLS based DIABLO method from the mixOmics R package^18^. The method extracts common variation across different data types through the selection of a subset of biomarkers across the Omics while discriminating between phenotypic, *i.e.,* in our instance T2D cases versus controls, groups. Feature pre-selection was performed using the *plsda* function of mixOmics R package, and the integrative analysis was done via the *block.splsda* function of the DIABLO algorithm. We used two PLS components for visualization of biomarkers and making predictions of T2D status on the test samples. The analysis workflow developed and used for our multiOmics data pre-processing and integration is available at https://github.com/NikolayOskolkov/IntegrativeOmicsWorkflow.

The multiple hold-out cross-validation procedure included 100 iterations. Instead of ranking based on loading scores, we required the features to appear in at least 70% of all train-test splits, to make the model generalizable and to provide a better weight between the expression, DNA methylation, and genotype Omics. Due to the low number of phenotype features, all were included in the integrative model.

### Ethical approval

Informed consent was obtained from donors of pancreatic islets or their relatives, and all procedures were approved by the Swedish Ethical Review Authority (Permit number 2011–263) in accordance with the Declaration of Helsinki.

### Supplementary Information

Supplementary Information 1.

Supplementary Information 2.

Supplementary Information 3.

Supplementary Table 1.

Supplementary Table 2.

## Data Availability

The human islet DNA methylation, RNA-seq and SNPs datasets generated for this study were deposited in the LUDC repository (https://www.ludc.lu.se/resources/repository, EPIC DNA methylation data, accession numbers LUDC2022.05.011, RNA-seq, accession number LUDC2022.05.013, and SNPs GWAS data, accession number LUDC2023.11.1). Data are available upon request through https://www.ludc.lu.se/resources/repository and jasmina.kravic@med.lu.se. Individual level data from the human pancreatic islets are not publicly available due to ethical and legal restrictions related to the Swedish Biobanks in Medical Care Act, the Personal Data Act and European Union’s General Data Protection Regulation and Data Protection Act. Data code generated during the current study is described under Statistical Analysis and Bioinformatic Tools and it is available at https://github.com/NikolayOskolkov/IntegrativeOmicsWorkflow.

## References

[1] Nasykhova YA, Barbitoff YA, Serebryakova EA, Katserov DS, Glotov AS (2019). Recent advances and perspectives in next generation sequencing application to the genetic research of type 2 diabetes. World J. Diabetes.

[2] Suzuki, K. *et al.* Multi-ancestry genome-wide study in >2.5 million individuals reveals heterogeneity in mechanistic pathways of type 2 diabetes and complications. *medRxiv* (2023). 10.1101/2023.03.31.23287839

[3] Bacos, K. *et al.* Type 2 diabetes candidate genes, including PAX5, cause impaired insulin secretion in human pancreatic islets. *J Clin Invest* **133** (2023). 10.1172/JCI163612 10.1172/JCI163612 PMC9927941 36656641

[4] Segerstolpe A (2016). Single-cell transcriptome profiling of human pancreatic islets in health and type 2 diabetes. Cell Metab..

[5] Lawlor N (2017). Single-cell transcriptomes identify human islet cell signatures and reveal cell-type-specific expression changes in type 2 diabetes. Genome Res..

[6] Wigger L (2021). Multi-omics profiling of living human pancreatic islet donors reveals heterogeneous beta cell trajectories towards type 2 diabetes. Nat. Metab..

[7] Xin Y (2016). RNA sequencing of single human islet cells reveals type 2 diabetes genes. Cell. Metab..

[8] Dayeh T (2014). Genome-wide DNA methylation analysis of human pancreatic islets from type 2 diabetic and non-diabetic donors identifies candidate genes that influence insulin secretion. PLoS Genet.

[9] Volkov P (2017). Whole-genome bisulfite sequencing of human pancreatic islets reveals novel differentially methylated regions in type 2 diabetes pathogenesis. Diabetes.

[10] Olsson AH (2014). Genome-wide associations between genetic and epigenetic variation influence mRNA expression and insulin secretion in human pancreatic islets. PLoS Genet.

[11] Ronn T (2023). Genes with epigenetic alterations in human pancreatic islets impact mitochondrial function, insulin secretion, and type 2 diabetes. Nat. Commun..

[12] Dayeh TA (2013). Identification of CpG-SNPs associated with type 2 diabetes and differential DNA methylation in human pancreatic islets. Diabetologia.

[13] Manolio TA (2009). Finding the missing heritability of complex diseases. Nature.

[14] Artzi NS (2020). Prediction of gestational diabetes based on nationwide electronic health records. Nat. Med..

[15] Holmgren G, Andersson P, Jakobsson A, Frigyesi A (2019). Artificial neural networks improve and simplify intensive care mortality prognostication: a national cohort study of 217,289 first-time intensive care unit admissions. J. Intensive Care.

[16] Tomasev N (2019). A clinically applicable approach to continuous prediction of future acute kidney injury. Nature.

[17] Rohart F, Gautier B, Singh A, Le Cao KA (2017). mixOmics: an R package for 'omics feature selection and multiple data integration. PLoS Comput Biol.

[18] Singh A (2019). DIABLO: an integrative approach for identifying key molecular drivers from multi-omics assays. Bioinformatics.

[19] Bouhaddani, S. E. *et al.* Evaluation of O2PLS in Omics data integration. *BMC Bioinformatics* **17 Suppl 2**, 11 (2016). 10.1186/s12859-015-0854-z 10.1186/s12859-015-0854-z PMC4959391 26822911

[20] Scutari M (2017). Bayesian network constraint-based structure learning algorithms: parallel and optimized implementations in the bnlearn R package. J. Stat. Softw..

[21] Acharjee A, Kloosterman B, Visser RG, Maliepaard C (2016). Integration of multi-omics data for prediction of phenotypic traits using random forest. BMC Bioinform..

[22] Argelaguet R, Velten B, Arnol D, Dietrich S, Zenz T, Marioni JC, Buettner F, Huber W, Stegle O (2018). Multi-omics factor analysis—a framework for unsupervised integration of multi-omics data sets. Mol. Syst. Biol..

[23] Wang B (2014). Similarity network fusion for aggregating data types on a genomic scale. Nat. Methods.

[24] Miotto R, Li L, Kidd BA, Dudley JT (2016). Deep patient: an unsupervised representation to predict the future of patients from the electronic health records. Sci. Rep..

[25] McInnes, H., Melville. UMAP: Uniform Manifold Approximation and Projection for Dimension Reduction. *arXiv***1802.03426v3** (2020).

[26] Tenenhaus A (2014). Variable selection for generalized canonical correlation analysis. Biostatistics.

[27] Altman N, Krzywinski M (2018). The curse(s) of dimensionality. Nat. Methods.

[28] Clarke R (2008). The properties of high-dimensional data spaces: implications for exploring gene and protein expression data. Nat. Rev. Cancer.

[29] Tibshirani R (1996). Regression shrinkage and selection via the lasso. J. Royal Stat. Soc..

[30] Fisher R (1936). The use of multiple measurements in taxonomic problems. Ann. Eugenics.

[31] Hall E (2014). Sex differences in the genome-wide DNA methylation pattern and impact on gene expression, microRNA levels and insulin secretion in human pancreatic islets. Genome Biol..

[32] Ohlson LO (1988). Risk factors for type 2 (non-insulin-dependent) diabetes mellitus. Thirteen and one-half years of follow-up of the participants in a study of Swedish men born in 1913. Diabetologia.

[33] Chicco D, Jurman G (2023). The Matthews correlation coefficient (MCC) should replace the ROC AUC as the standard metric for assessing binary classification. BioData Min..

[34] Schrader S (2022). Novel subgroups of type 2 diabetes display different epigenetic patterns, which associate with future diabetic complications. Diabetes Care.

[35] Sansbury FH (2012). SLC2A2 mutations can cause neonatal diabetes, suggesting GLUT2 may have a role in human insulin secretion. Diabetologia.

[36] Ji H (2017). Elevated OPRD1 promoter methylation in Alzheimer's disease patients. PLoS ONE.

[37] Bacos K (2016). Blood-based biomarkers of age-associated epigenetic changes in human islets associate with insulin secretion and diabetes. Nat. Commun..

[38] Lawlor N, Marquez EJ, Orchard P, Narisu N, Shamim MS, Thibodeau A, Varshney A, Kursawe R, Erdos MR, Kanke M, Gu H (2019). Multiomic profiling identifies cis-regulatory networks underlying human pancreatic β cell identity and function. Cell Rep..

[39] Hall E (2018). The effects of high glucose exposure on global gene expression and DNA methylation in human pancreatic islets. Mol. Cell. Endocrinol..

[40] Thielen L, Shalev A (2018). Diabetes pathogenic mechanisms and potential new therapies based upon a novel target called TXNIP. Curr. Opin. Endocrinol. Diabetes Obes..

[41] Bradshaw TY (2016). A reduction in Drp1-mediated fission compromises mitochondrial health in autosomal recessive spastic ataxia of Charlevoix Saguenay. Hum. Mol. Genet..

[42] Ling C (2008). Epigenetic regulation of PPARGC1A in human type 2 diabetic islets and effect on insulin secretion. Diabetologia.

[43] Olsson AH (2011). Decreased expression of genes involved in oxidative phosphorylation in human pancreatic islets from patients with type 2 diabetes. Eur. J. Endocrinol..

[44] Crist RC, Clarke TK (2018). OPRD1 genetic variation and human disease. Handb. Exp. Pharmacol..

[45] Kvaloy K, Kulle B, Romundstad P, Holmen TL (2013). Sex-specific effects of weight-affecting gene variants in a life course perspective–The HUNT Study. Norway. Int. J. Obes. (Lond).

[46] Winkler TW (2018). A joint view on genetic variants for adiposity differentiates subtypes with distinct metabolic implications. Nat. Commun..

[47] Mendelson MM (2017). Association of body mass index with dna methylation and gene expression in blood cells and relations to cardiometabolic disease: a mendelian randomization approach. PLoS Med..

[48] Wahl S (2017). Epigenome-wide association study of body mass index, and the adverse outcomes of adiposity. Nature.

[49] Ervin JF (2007). Postmortem delay has minimal effect on brain RNA integrity. J. Neuropathol. Exp. Neurol..

[50] Hall E (2019). Glucolipotoxicity alters insulin secretion via epigenetic changes in human islets. Diabetes.

[51] Yang BT (2011). Insulin promoter DNA methylation correlates negatively with insulin gene expression and positively with HbA(1c) levels in human pancreatic islets. Diabetologia.

[52] Yang BT (2012). Increased DNA methylation and decreased expression of PDX-1 in pancreatic islets from patients with type 2 diabetes. Mol. Endocrinol..

[53] Du P, Kibbe WA, Lin SM (2008). lumi: a pipeline for processing Illumina microarray. Bioinformatics.

[54] Liu J, Siegmund KD (2016). An evaluation of processing methods for HumanMethylation450 BeadChip data. BMC Genom..

[55] Du P (2010). Comparison of Beta-value and M-value methods for quantifying methylation levels by microarray analysis. BMC Bioinform..

[56] Patro R, Duggal G, Love MI, Irizarry RA, Kingsford C (2017). Salmon provides fast and bias-aware quantification of transcript expression. Nat. Methods.

[57] Love MI, Huber W, Anders S (2014). Moderated estimation of fold change and dispersion for RNA-seq data with DESeq2. Genome Biol..

[58] Turner S, Armstrong LL, Bradford Y, Carlson CS, Crawford DC, Crenshaw AT, de Andrade M, Doheny KF, Haines JL, Hayes G, Jarvik G (2011). Quality control procedures for genome-wide association studies. Curr. Protocols Hum. Gene..

[59] Purcell S (2007). PLINK: a tool set for whole-genome association and population-based linkage analyses. Am. J. Hum. Genet..

