# Supplementary material for: Predicting type 2 diabetes via machine learning integration of multiple omics from human pancreatic islets
Source: Sci Rep. 2024 Jun 25;14:14637. doi: 10.1038/s41598-024-64846-3 (PMC11199577; doi:10.1038/s41598-024-64846-3)
Supplement: Supplementary file 1 — Supplementary Information 1. [file 41598_2024_64846_MOESM1_ESM.docx]

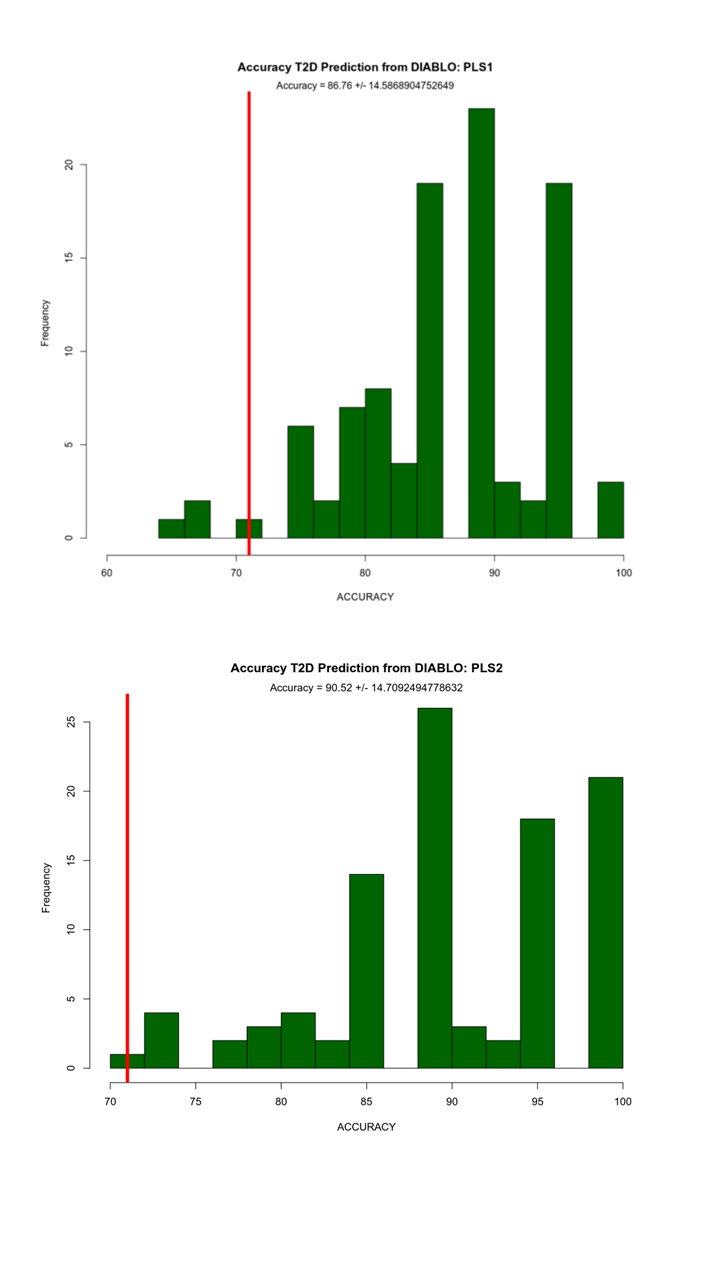
**Supplementary Figure 1.** Histogram of T2D prediction accuracy values from the integrative multiOmics DIABLO model for 100 train-test splits of the multiOmics dataset of 110 individuals (32 T2D cases and 78 controls) within the multiple hold-out cross-validation framework. The figure demonstrates that the DIABLO model significantly outperforms a baseline 71% prediction accuracy that can be expected from the imbalanced classification task. The red line depicts the naïve baseline model.
