# Supplementary material for: Predicting type 2 diabetes via machine learning integration of multiple omics from human pancreatic islets
Source: Sci Rep. 2024 Jun 25;14:14637. doi: 10.1038/s41598-024-64846-3 (PMC11199577; doi:10.1038/s41598-024-64846-3)
Supplement: Supplementary file 2 — Supplementary Information 2. [file 41598_2024_64846_MOESM2_ESM.docx]

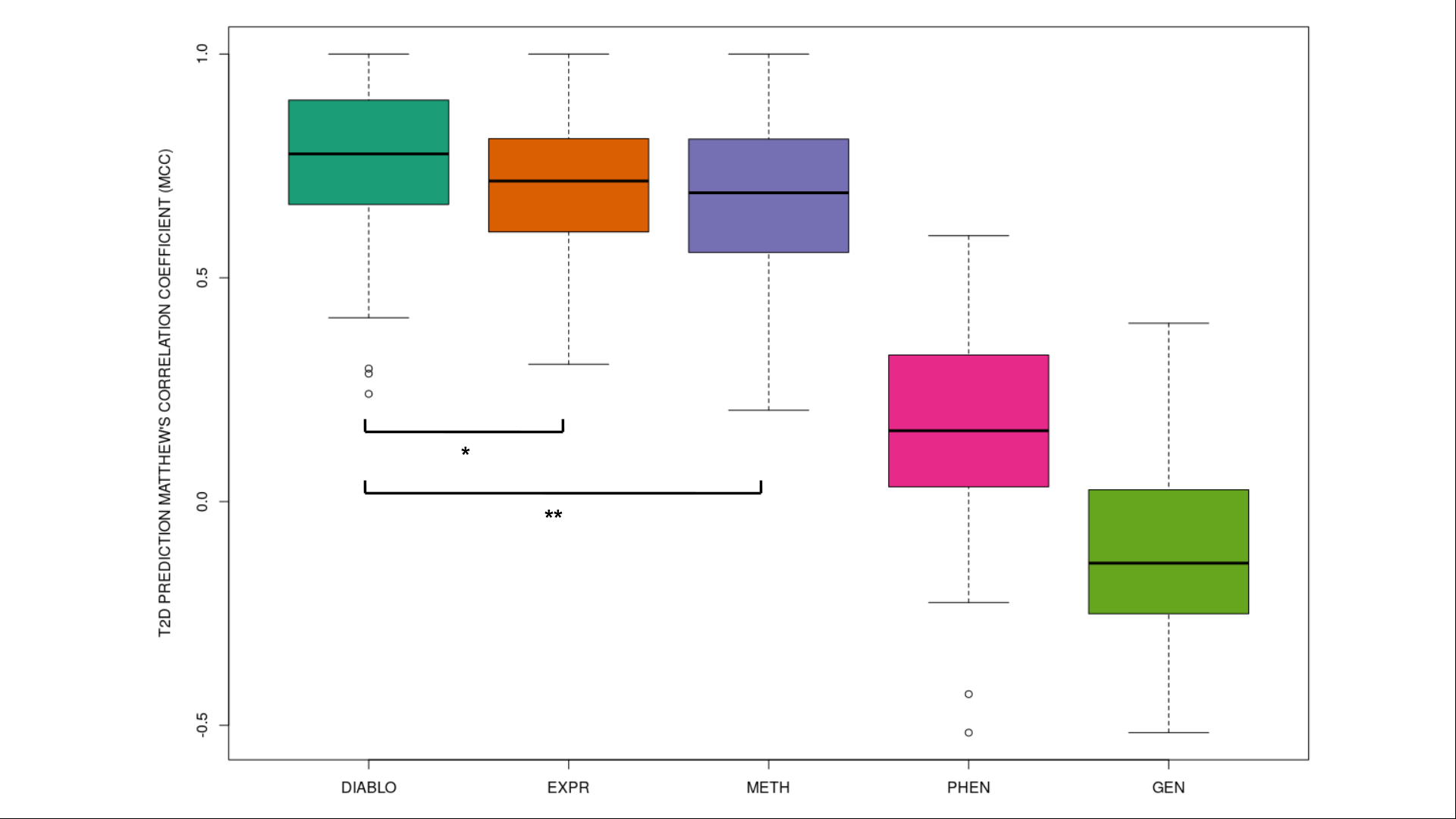


**Supplementary Figure 2.** Comparison of T2D prediction by the integrative multiOmics DIABLO model versus the PLS-DA models on the three individual Omics data sets (mRNA expression (exp), DNA methylation (meth), and genetic variation/SNPs (gen)) and the clinical phenotypes (phen) in terms of Matthew’s Correlation Coefficient (MCC). Sig. *: Mann-Whittney U test p-value = 0.03, Sig. ***: Mann-Whittney U test p-value = 0.002
