# Supplementary material for: Predicting type 2 diabetes via machine learning integration of multiple omics from human pancreatic islets
Source: Sci Rep. 2024 Jun 25;14:14637. doi: 10.1038/s41598-024-64846-3 (PMC11199577; doi:10.1038/s41598-024-64846-3)

**Supplementary Figure 3.** Network plot: feature correlations for each pair of Omics. **a**) gene expression–DNA methylation (8 edges per node on average), **b**) gene expression-genotype (1 edge per node on average), **c**) gene expression-phenotype (1 edge per node on average), **d**) genotype-DNA methylation (1 edge per node on average), **e**) phenotype-DNA methylation (1 edge per node on average) and **f**) genotype-phenotype (2 edges per node on average).


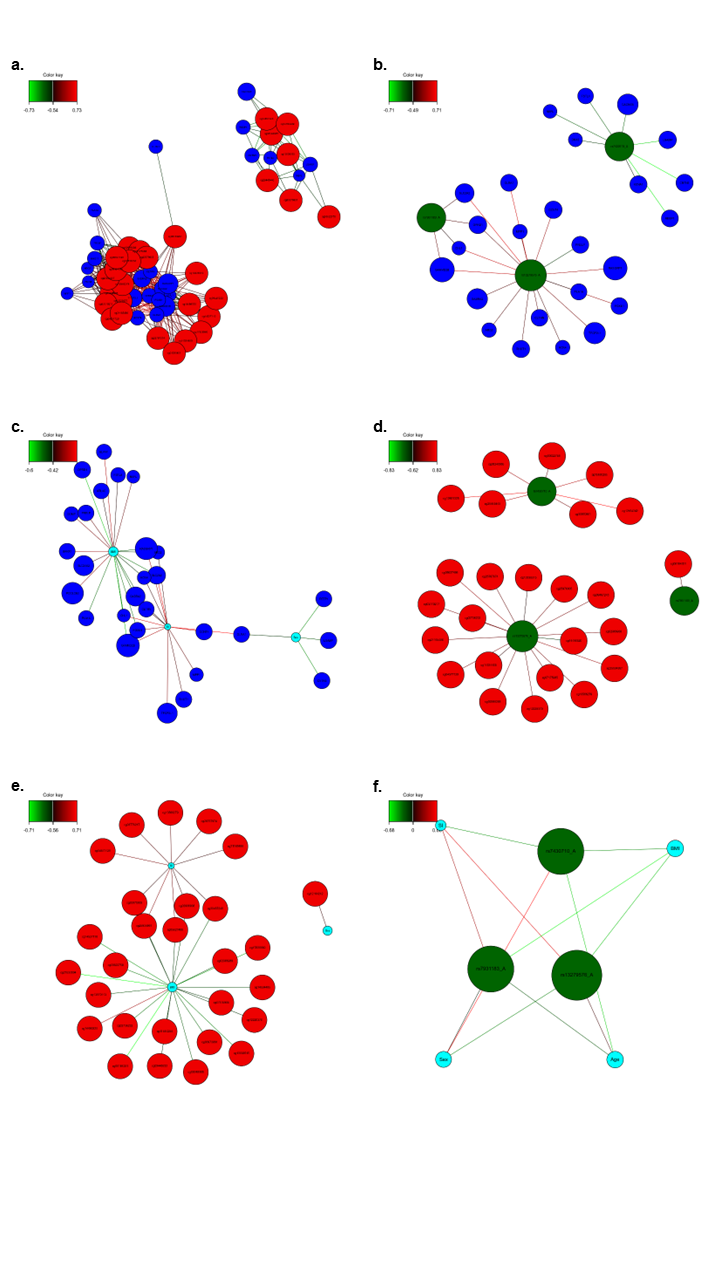

Supplement: Supplementary file 3 — Supplementary Information 3. [file 41598_2024_64846_MOESM3_ESM.docx]
